# Supplementary material for: Top-down acetylcholine signaling via olfactory bulb vasopressin cells contributes to social discrimination in rats
Source: Commun Biol. 2021 May 21;4:603. doi: 10.1038/s42003-021-02129-7 (PMC8140101; doi:10.1038/s42003-021-02129-7)
Supplement: Supplementary file 2 — Supplementary Information [file 42003_2021_2129_MOESM2_ESM.pdf]

Supplementary Information

Title

Top-down acetylcholine signaling via olfactory bulb vasopressin cells contributes to social discrimination in rats

Authors

Hajime Suyama[1], Veronica Egger[1], Michael Lukas[1]

[1]Institute of Zoology, Neurophysiology, University of Regensburg, 93040 Regensburg, Germany

Corresponding author

Michael Lukas

Institute of Zoology, Neurophysiology, University of Regensburg, 93040 Regensburg, Germany

e-mail: michael.lukas@ur.de

Supplementary Fig. 1 **VPC activation in the MOB and AOB**

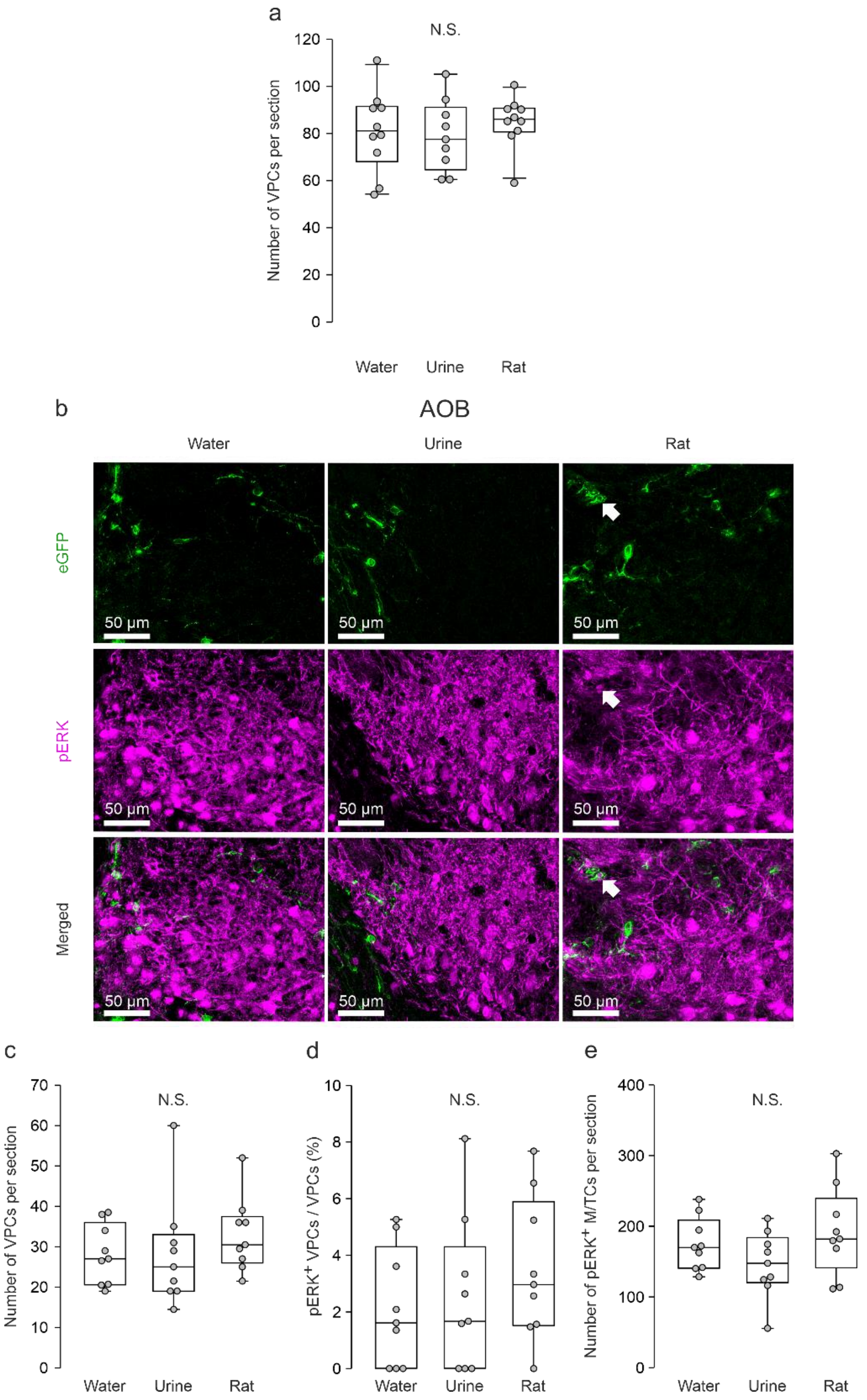

(a) Averaged number of MOB VPCs per section in different stimulation groups. n=10 rats (water), n=9 rats (urine), n=10 rats (rat). Data are presented as box-plots including first, median, and third quartiles with whiskers representing the range of data points and distribution of single data points. One-way ANOVA, N.S., not significant. (b) Representative average z-projections of the accessory olfactory bulb that were immune-stained for eGFP (green, CF488) and pERK (magenta, CF 594) following water, urine, or rats stimulation. Arrows indicate a cell that is double-labeled for eGFP and pERK. Scale bar, 50 µm valid for all images in the panel. (c) Averaged number of AOB VPCs per section in different stimulation groups. (d) Averaged fraction of pERK$^+$ AOB VPCs of all AOB VPCs in different stimulation groups (%). (e) Averaged number of pERK$^+$ AOB M/TCs per section in different stimulation groups. Data are presented as box-plots including first, median, and third quartiles with whiskers representing the range of data points and distribution of single data points. One-way ANOVA, N.S., not significant. n=9 rats (water), n=9 rats (urine), n=9 rats (rat).

Supplementary Fig. 2 **ACh does not alter intrinsic excitability in VPCs and electrical AOB stimulation does not evoke excitatory responses in MOB VPCs**

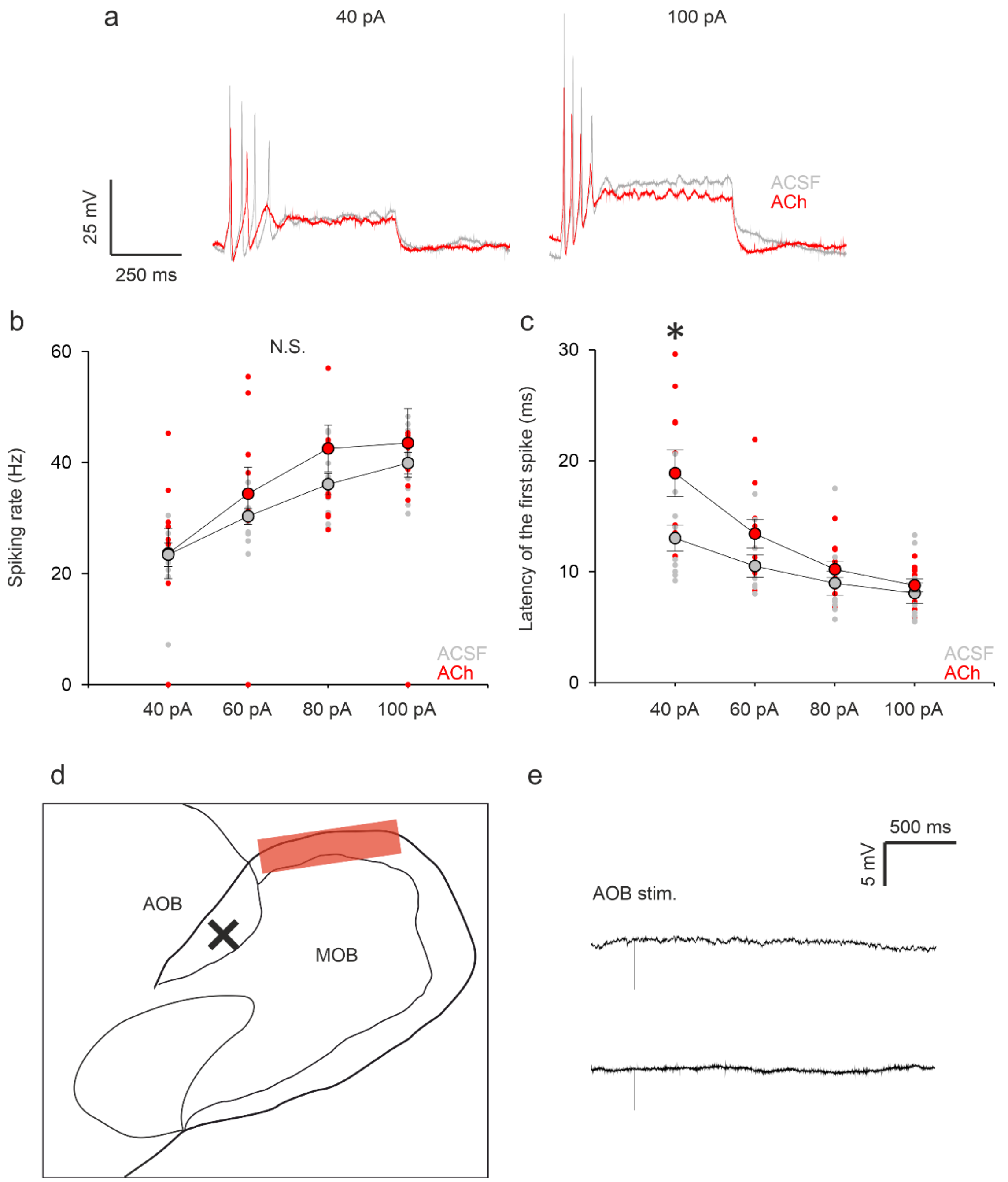

(a) Representative traces of responses to somatically applied current steps in the ACSF condition (grey) and during bath application of ACh (100 µM, red). (b+c) Spiking rates of action potential trains and latency of the first spike evoked by somatic current injection (40-100 pA) in the ACSF condition (grey) and during bath application of ACh (100 µM, red). (2) × (2) mixed model ANOVA (intensity [within subject] × treatment [within-subject]). N.S., not significant. LSD for single comparison, *$p<0.05$ ACh (40pA) vs. ACSF (40pA). Data are means ± SEM including distribution of single data points. n=10 cells. (d) Schematic drawing of the sagittal OB. The cross indicates where the stimulation electrode was positioned. The red bar indicates the dorsal region of the MOB where patch-clamp recordings from MOB VPCs were performed. (e) Representative averaged trace of response from MOB VPCs to electrical vomeronasal nerve/ AOB stimulation (50-500 µA, 100 µs).

Supplementary Fig. 3 **Intracellular calcium indicator does not alter ACh effects on evoked PSP amplitudes in VPCs**

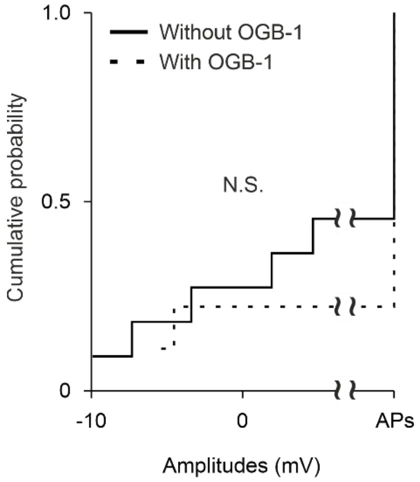

Cumulative probability of evoked PSP amplitudes in the ACh condition with or without OGB-1 in the intracellular solution (n=9/11 cells). The amplitudes of APs were set as 100 mV. Kruskal-Wallis test for variation comparison. N.S., not significant.

Supplementary Fig. 4 **Atropine and VP microinjection into the OB does not interfere with non-social investigatory/play behavior and habituation.**

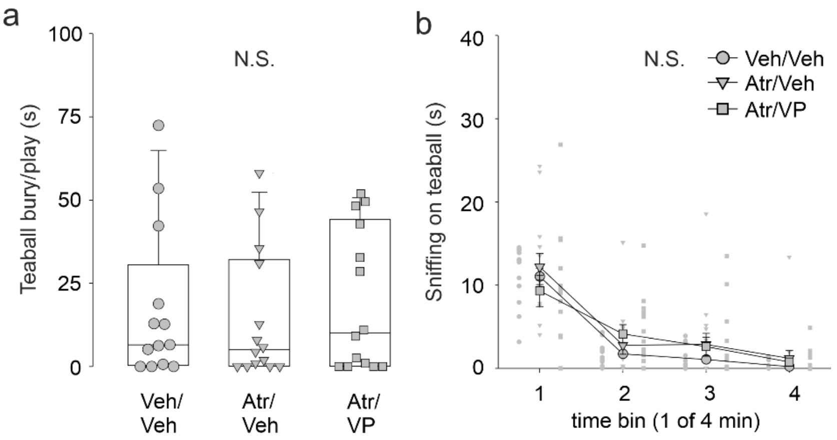

(a) Amount of time (in s) that rats are engaged in burying/playing with the teaball during neutral odor stimulation (amylacetate or carvone). Data are presented as box-plots including first, median, and third quartiles with whiskers representing the range of data points and distribution of single data points. Kruskal-Wallis Test, n=13 rats (Veh/Veh), n=14 rats (Atr/Veh, 1µg), n=14 rats (Atr/VP, 1µg/1ng). (b) Amount of time (in s) within time bins of 1 min rats investigate the teaball during neutral odor presentation. Data are presented as means ± SEM including distribution of single data points. (4) × (3) mixed model ANOVA (time bin [within-subject] × treatment [between-subject]), n=13 rats (Veh/Veh), n=14 rats (Atr/Veh, 1µg), n=14 rats (Atr/VP, 1 µg/1ng). N.S., not significant.
