# Supplementary material for: Top-down acetylcholine signaling via olfactory bulb vasopressin cells contributes to social discrimination in rats
Source: Commun Biol. 2021 May 21;4:603. doi: 10.1038/s42003-021-02129-7 (PMC8140101; doi:10.1038/s42003-021-02129-7)
Supplement: Supplementary file 3 — Descriptions of Additional Supplementary Files [file 42003_2021_2129_MOESM3_ESM.pdf]

Descriptions of Additional Supplementary Files

Supplementary data 1
Description: All source data underlying the graphs and charts presented in the main figures.
