# Supplementary material for: Top-down acetylcholine signaling via olfactory bulb vasopressin cells contributes to social discrimination in rats
Source: Commun Biol. 2021 May 21;4:603. doi: 10.1038/s42003-021-02129-7 (PMC8140101; doi:10.1038/s42003-021-02129-7)
Supplement: Supplementary file 5 — Reporting Summary [file 42003_2021_2129_MOESM5_ESM.pdf]

nature research

Corresponding author(s): Michael Lukas

Last updated by author(s): Apr 6, 2021

Reporting Summary

Nature Research wishes to improve the reproducibility of the work that we publish. This form provides structure for consistency and transparency in reporting. For further information on Nature Research policies, see our Editorial Policies and the Editorial Policy Checklist.

Statistics

For all statistical analyses, confirm that the following items are present in the figure legend, table legend, main text, or Methods section.

n/a	Confirmed	
[ ]	[x]	The exact sample size (*n*) for each experimental group/condition, given as a discrete number and unit of measurement
[ ]	[x]	A statement on whether measurements were taken from distinct samples or whether the same sample was measured repeatedly
[ ]	[x]	The statistical test(s) used AND whether they are one- or two-sided<br>*Only common tests should be described solely by name; describe more complex techniques in the Methods section.*
[ ]	[x]	A description of all covariates tested
[ ]	[x]	A description of any assumptions or corrections, such as tests of normality and adjustment for multiple comparisons
[ ]	[x]	A full description of the statistical parameters including central tendency (e.g. means) or other basic estimates (e.g. regression coefficient) AND variation (e.g. standard deviation) or associated estimates of uncertainty (e.g. confidence intervals)
[ ]	[x]	For null hypothesis testing, the test statistic (e.g. *F*, *t*, *r*) with confidence intervals, effect sizes, degrees of freedom and *P* value noted<br>*Give P values as exact values whenever suitable.*
[x]	[ ]	For Bayesian analysis, information on the choice of priors and Markov chain Monte Carlo settings
[x]	[ ]	For hierarchical and complex designs, identification of the appropriate level for tests and full reporting of outcomes
[ ]	[x]	Estimates of effect sizes (e.g. Cohen's *d*, Pearson's *r*), indicating how they were calculated

Our web collection on statistics for biologists contains articles on many of the points above.

Software and code

Policy information about availability of computer code

Data collection	Fluorescent images of the stained slices were obtained using the software, LAS X (LEICA, Wetzlar, Germany). Tile-stitching and z-stack of pictures were performed by LAS X (LEICA, Wetzlar, Germany). After taking pictures, those were processed by 3D deconvolution or the Lightning & Thunder process (LAS X) to improve the contrast. Pictures were converted to .tif files using Fiji (ImageJ, downloaded from https://imagej.net/Fiji/Downloads).<br>Electrophysiological recordings were performed using an EPC-10 and Patchmaster software v2x73.5 (HEKA, Lambrecht, Germany).<br>Calcium Imaging was recorded by two-photon laser scanning microscopy on a Femto-2D microscope (Femtonics, Budapest, Hungary) using MES v4.5.613 software (Femtonics).
Data analysis	Immunoreactive cells were counted manually using the multi-point tool or cell counter plug-in in Fiji (ImageJ, downloaded from https://imagej.net/Fiji/Downloads).<br>Electrophysiology was analysed using Origin 2018b (Origin Lab Corporation, Northampton, MA, USA).<br>The behavioral analysis was done using JWatcher (downloaded from https://www.jwatcher.ucla.edu/).<br>Statistics were performed with SPSS (ver. 26, IBM, Armonk, NY, USA) and G*Power (ver. 3.1.9.2, Franz Faul, University of Kiel).

For manuscripts utilizing custom algorithms or software that are central to the research but not yet described in published literature, software must be made available to editors and reviewers. We strongly encourage code deposition in a community repository (e.g. GitHub). See the Nature Research guidelines for submitting code & software for further information.

Data

Policy information about availability of data

All manuscripts must include a data availability statement. This statement should provide the following information, where applicable:

- Accession codes, unique identifiers, or web links for publicly available datasets
- A list of figures that have associated raw data
- A description of any restrictions on data availability

All numerical data that is represented in the figures (Figs. 1-5, Supplementary Figs. 1-4) is available as source data file corresponding to the figures (Supplementary Data 1). All other data are available from the corresponding author (or other sources, as applicable) on reasonable request.

Field-specific reporting

Please select the one below that is the best fit for your research. If you are not sure, read the appropriate sections before making your selection.

☒ Life sciences ☐ Behavioural & social sciences ☐ Ecological, evolutionary & environmental sciences

For a reference copy of the document with all sections, see nature.com/documents/nr-reporting-summary-flat.pdf

Life sciences study design

All studies must disclose on these points even when the disclosure is negative.

Sample size	Sample size was determined to be adequate based on the magnitude of measurable effects between groups in the main command variable of the different experiments. For confirmation please see the achieved statistical power of statistical tests in Table 1.
Data exclusions	In the neuronal activation experiments (Fig. 1) animals were excluded that did not have direct nose-contact with the stimulus (rat, urine, or water). Also, 2 animals in are not included in the analysis of the AOB experiment (Suppl. Fig. 1) as the AOB was not visible on the corresponding brain slices. The HDB experiment (Fig. 5) was only done in the last cohort of stimulation experiments as in the other cohorts only the olfactory bulbs, but not the HDB was collected. No anatomical outliers in stereotaxic cannula implantation had to be excluded from behavioral analysis (Fig. 6). However, one animal from the control group was excluded, as it showed a high amount of unspecific sexual/ mounting behavior towards the stimulus animals throughout the sampling as well as the discrimination phase (Engelmann et al., Nat Protoc 6, 2011). Electrophysiology experiments were only performed in case the patched cells had a holding current below ~ -50 pA and no drastic drift in the resting membrane potential. In electrophysiology and calcium imaging experiments only cells that did not die (holding current > -200pA) before the completion of the planned experimental measurements were included in the analysis.
Replication	All behavioral pharmacology (Fig. 6) and neuronal activity (Fig. 1+5, Suppl. Fig. 1) experiments in the manuscript were performed in 3 cohorts. Thereby demonstrating replication and homogenous effects of olfactory stimulation and pharmacology. Moreover, all cohorts tested for pharmacological experiments showed homogenous effects of the drug although the baseline levels of investigation slightly differed from one cohort to the other. To illustrate replication in acute in-vitro slice experiments it is clearly indicated in the results section from how many different animals the different repetitions/measurements originate from.
Randomization	Rats in immunohistochemistry and behavioral pharmacology experiments were randomized in the experimental groups. In case rats from the same litter were used, they were evenly distributed between experimental groups in order to increase the diversity within a group. Pharmacological treatment during electrophysiological recordings from acute olfactory bulb slices was performed using a within-subject design. Also, these experiments were performed over several months to years in different individuals from different litters. Therefore, the chose of pharmacological treatment was also randomized.
Blinding	All the data was analyzed by an observer blinded with respect to groups and pharmacology.

Reporting for specific materials, systems and methods

We require information from authors about some types of materials, experimental systems and methods used in many studies. Here, indicate whether each material, system or method listed is relevant to your study. If you are not sure if a list item applies to your research, read the appropriate section before selecting a response.

Materials & experimental systems

n/a	Involved in the study	
☐	☒	Antibodies
☒	☐	Eukaryotic cell lines
☒	☐	Palaeontology and archaeology
☐	☒	Animals and other organisms
☒	☐	Human research participants
☒	☐	Clinical data
☒	☐	Dual use research of concern

Methods

n/a	Involved in the study	
☒	☐	ChIP-seq
☒	☐	Flow cytometry
☒	☐	MRI-based neuroimaging

Antibodies

Antibodies used	Primary antibodies were goat anti-GFP (1:1000, #600-101-215S, Rockland, Limerick, PA, USA), Rabbit anti-P-p44/42 MAPK (1:1000, #9101S, Cell Signaling Technology, Frankfurt am Main, Germany), Sheep Anti-Choline Acetyltransferase (1:125, ab18736, Abcam, Berlin, Germany).
Validation	All antibodies were validated by the manufacturer or publications:<br>goat anti-GFP (Reactivity: wt, rGFP, eGFP; Purity And Specificity: GFP antibody was prepared from monospecific antiserum by immunoaffinity chromatography using Green Fluorescent Protein (Aequorea victoria) coupled to agarose beads followed by solid phase adsorption(s) to remove any unwanted reactivities. Assay by immunoelectrophoresis resulted in a single precipitin arc against anti-Goat Serum and purified and partially purified Green Fluorescent Protein (Aequorea victoria). No reaction was observed against Human, Mouse or Rat serum proteins)<br>Rabbit anti-P-p44/42 MAPK (Reactivity: Human, Mouse, Rat, Hamster, Monkey, Mink, D. melanogaster, Zebrafish, Bovine, Pig, C. elegans. Species reactivity is determined by testing in at least one approved application (e.g., western blot); Specificity/Sensitivity: Phospho-p44/42 MAPK (Erk1/2) (Thr202/Tyr204) Antibody detects endogenous levels of p44 and p42 MAP Kinase (Erk1 and Erk2) when phosphorylated either individually or dually at Thr202 and Tyr204 of Erk1 (Thr185 and Tyr187 of Erk2). The antibody does not cross-react with the corresponding phosphorylated residues of either JNK/SAPK or p38 MAP Kinase, and does not cross-react with non-phosphorylated Erk1/2.)<br>Sheep Anti-Choline Acetyltransferase (Reactivity: Rat, Pig; Reference: Nature Neuroscience volume 22, pages524–528(2019), PLoS One 2013;8(1):e53814)

Animals and other organisms

Policy information about studies involving animals; ARRIVE guidelines recommended for reporting animal research

Laboratory animals	Behavior: male Wistar rats (5-6 weeks).<br>Immunohistochemistry: male heterozygous VP-eGFP Wistar rats (5-6 weeks).<br>Simulus rats: male Wistar rats (3-4 weeks).<br>Electrophysiology: heterozygous VP-eGFP Wistar rats (11-18 days) of either sex.
Wild animals	Study did not involve wild animals.
Field-collected samples	Study did not involve samples collected from the wild
Ethics oversight	All experiments were conducted according to national and institutional guidelines for the care and use of laboratory animals, the rules laid down by the EC Council Directive (86/89/ECC) and German animal welfare. The study protocol was approved by the Government of Unterfranken (RUF-55.2.2-2532-2-539 and RUF-55.2.2-2532-2-1291).

Note that full information on the approval of the study protocol must also be provided in the manuscript.
